# Supplementary material for: The CDH1 c.1901C>T Variant: A Founder Variant in the Portuguese Population with Severe Impact in mRNA Splicing
Source: Cancers (Basel). 2021 Sep 4;13(17):4464. doi: 10.3390/cancers13174464 (PMC8430675; doi:10.3390/cancers13174464)
Supplement: Supplementary file 1 [file cancers-13-04464-s001.zip › Figures/Supplemental_Figure_S1.pdf]

Supplemental Figure S1.
Partial pedigrees with corresponding haplotypes for all families studied.

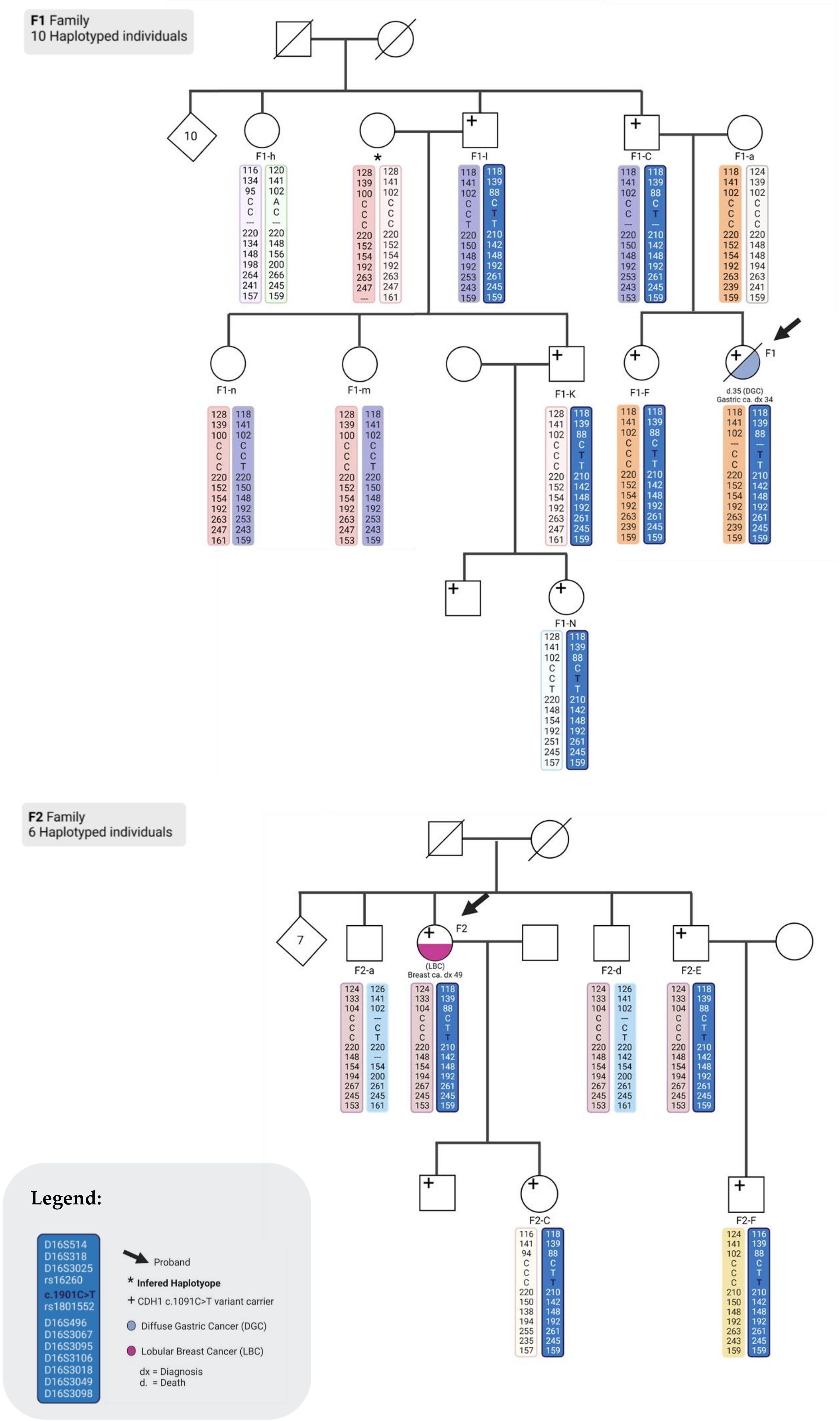

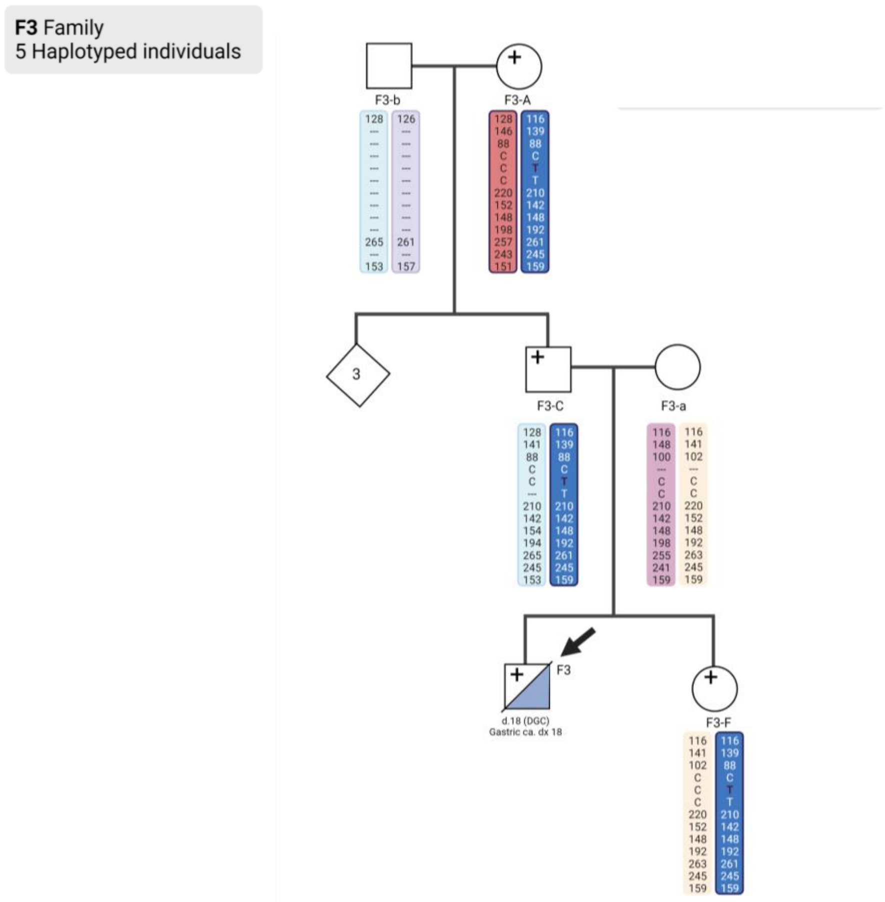

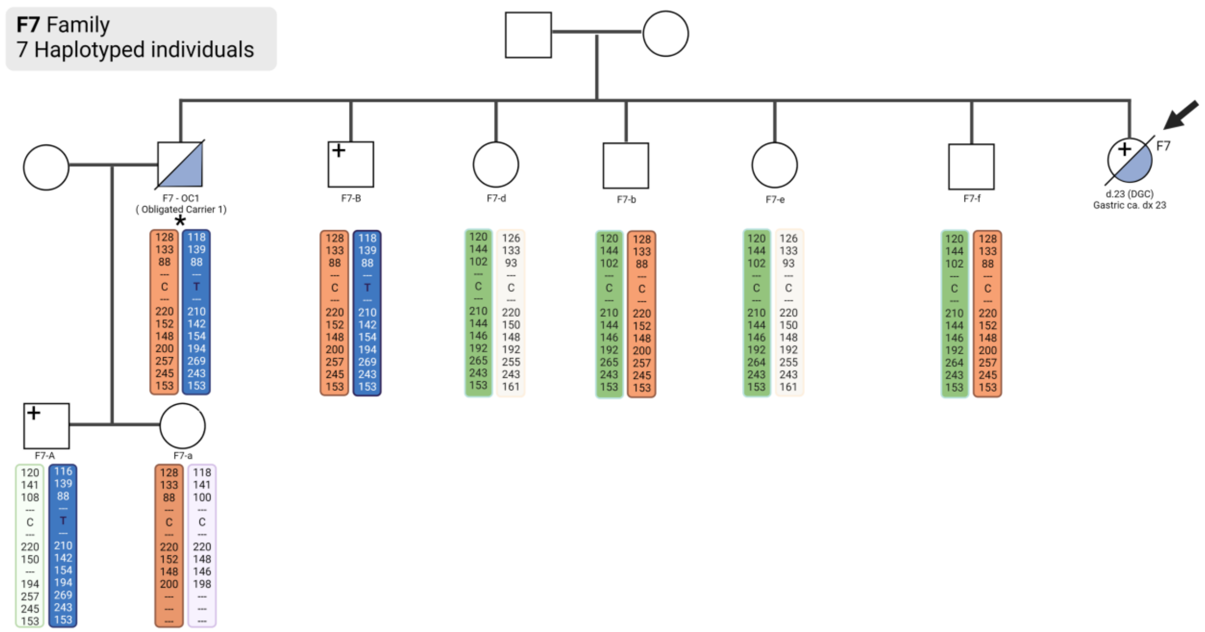

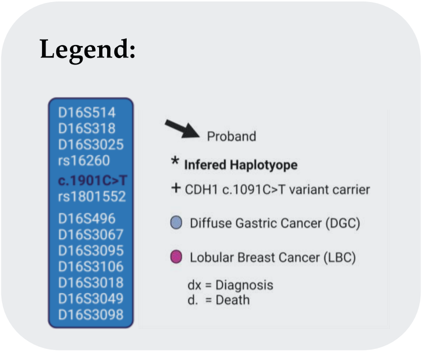

12 polymorphic markers used for F1, F2, F3 and F7 families. These families carrying the c.1901C>T mutation were the ones considered for the age mutation's estimation.

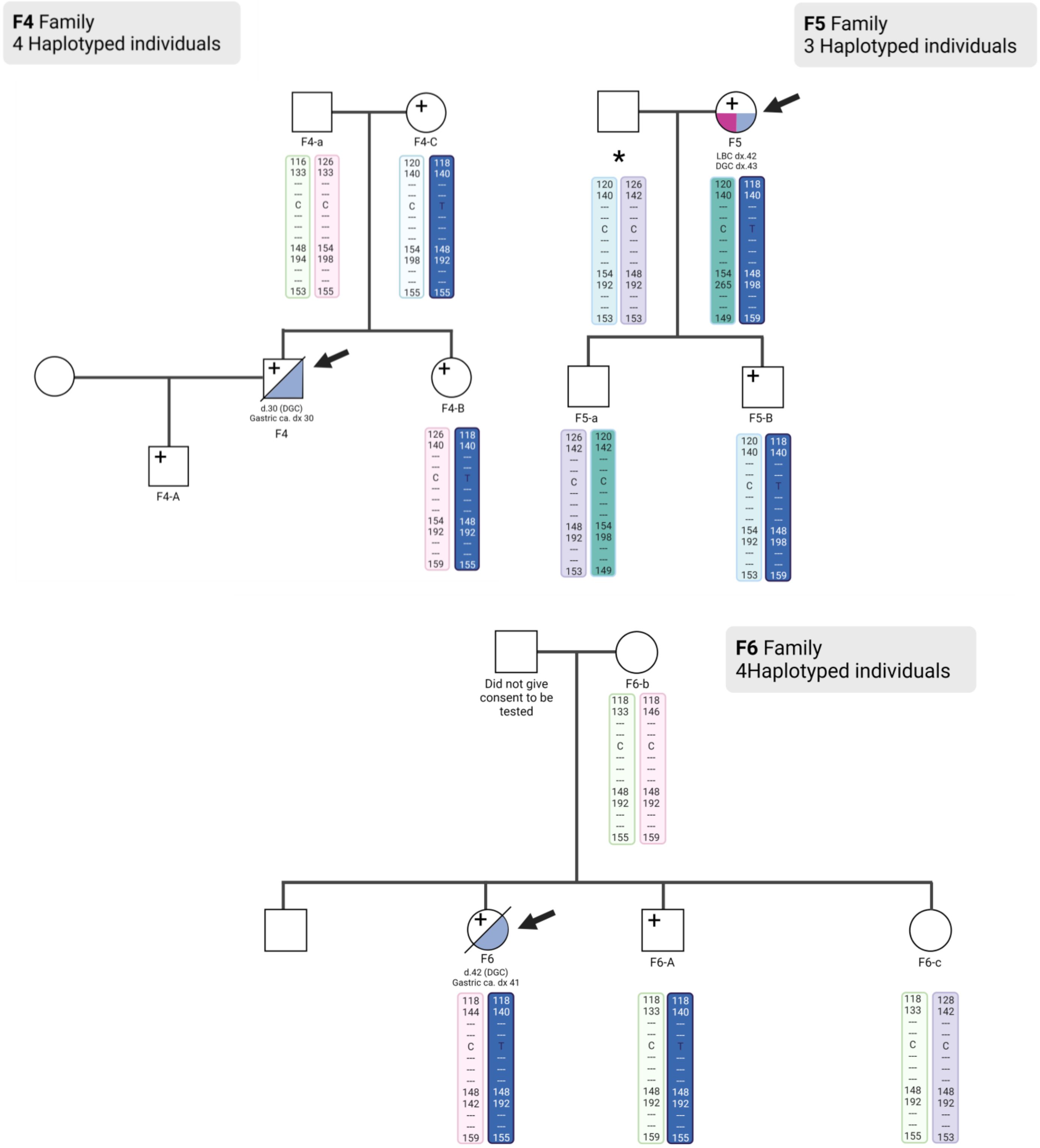

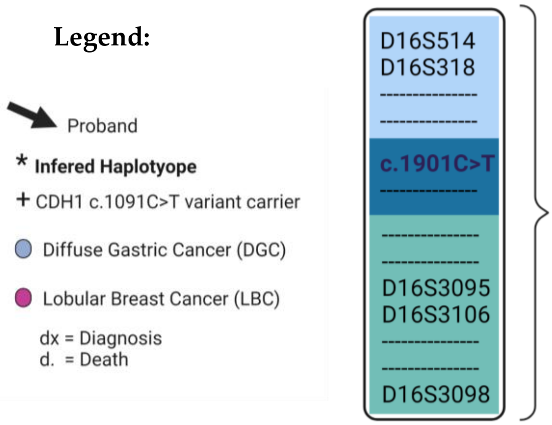

6 polymorphic markers used for F4, F5 and F6 families. Since we could not confidently identify the segregating haplotypes in these individuals, these families carrying the c.1901C>T mutation were not considered for the age mutation's estimation .
