# Supplementary material for: The CDH1 c.1901C>T Variant: A Founder Variant in the Portuguese Population with Severe Impact in mRNA Splicing
Source: Cancers (Basel). 2021 Sep 4;13(17):4464. doi: 10.3390/cancers13174464 (PMC8430675; doi:10.3390/cancers13174464)
Supplement: Supplementary file 1 [file cancers-13-04464-s001.zip › Tables/All_Supplemental_Tables_Vfinal.pdf]

Supplemental Table S1.

HDGC Clinical Criteria, updated in 2020.

HDCG Clinical Criteria
Family Criteria
families with ≥2 cases of GC regardless of age, with at least one **DGC**
≥1 case of **DGC** at any age, and ≥1 case of **LBC** at age <70 years, in different family members
≥2 cases of **LBC** in family members <50 years of age
Individual Criteria
Having **DGC** at age <50 years
Bilateral **LBC**, diagnosed at age <70 years
Having **DGC** and **LBC**, both diagnosed at age <70 years
**DGC** at any age in individuals with a personal or family history (1st degree relative) of cleft lip or cleft palate
**DGC** at any age in individuals of Māori ethnicity
Gastric in situ signet ring cells or pagetoid spread of signet ring cells in individuals <50 years of age

Supplemental Table S2.

List of primers and PCR conditions for colony-PCR strategy.

Technical Application		Primer's sequence	Conditions
**cDNA amplification/ sequencing**	ex11F	5′_CAGCACGTACACAGCCCTAA_3′	Initial denaturation step at 95 ºC for 15 minutes. Then, a touchdown from 65 ºC to 58ºC (5 cycles, -1ºC/cycle) with a denaturation step at 95 ºC for 30 seconds and extension at 72 ºC for 1 minute. In the next 30 cycles denaturation was done at 95 ºC for 30 seconds, annealing at 57 ºC for 1 minute and 30 seconds and extension for 1 minute. The final extension was done for 10 minutes at 72ºC.
	ex13R	5′_CCTCCAAGAATCCCCAGAATG_3′	
**Colony-PCR**	M13F	5′-GTAAAACGACGGCCAG-3′	After an initial 10 minutes denaturation step at 95ºC, a touchdown reaction using annealing temperatures between 55ºC and 50ºC (-1ºC/cycle) was performed, followed by 30 cycles with 50ºC as the annealing temperature. The final extension was done at 72ºC, for 10 minutes
	M13R	5′-CAGGAAACAGCTATGAC-3′	
**rs1801552 amplification (gDNA)**	ex13F	5′_CATACCTAAATAAAACCCAAGCAGCTC_3′	After initial denaturation step at 95 ºC for 15 minutes, a touchdown from 65 ºC to 62 ºC (6 cycles, -0,5 ºC/cycle) was done, with a denaturation step at 95 ºC for 30 seconds and extension at 72 ºC for 2 minutes and 30 seconds. In the next 29 cycles denaturation was done at 95 ºC for 30 seconds, annealing at 65 ºC for 1 minute and 30 seconds and extension for 2 minutes and 15 seconds. The final extension was done for 10 minutes at 72ºC.
	ex13R	5′_AACTTGGGAGTCTCTTTCCCACATC_3′	
**Snapshot**	ex12F	5′_GGAATCCAAAGCCTCAGGTC_3′	Initial 10 minutes denaturation at 95ºC, followed by a touchdown reaction with annealing temperature 62ºC to 56ºC (-1ºC/cycle), followed by 29 cycles of 56ºC annealing temperature. The final extension was done at 72ºC, for 10 minutes.
**Snapshot**	ex13R	5′_CCTCCAAGAATCCCCAGAATG_3′	
**Snapshot rs1801552**	SBE primer	5′_CTGTGCCTTCCTACAGACGCC_3′	The program consisted of 25 cycles with an initial denaturation step at 96ºC for 10 seconds, an annealing step at 56ºC for 5 seconds and an extension step of 30 seconds at 60ºC

Supplemental Table S3.

List of primers and PCR conditions for polymorphic marker studies.

Polymorphic marker	Distance to *CDH1*	Location		Primer Forward<br>Primer Reverse	Predicted size of the amplicon (bp)	PCR Conditions			
						Touchdown range(ºC) -1ºC/ cycle	Ta (ºC)	Program	
**D16S514**	6432933	62304172	62304292	5′_CTATCCACTCACTTTCCAGG_3′<br>5′_TCCCACTGATCATCTTCTC_3′	117 - 133	57ºC–53ºC	54ºC		
**D16S318**	1475206	67261888	67262019	5′_CTGTGGTGTCACATCAGGAA_3′<br>5′_GACTACACATGAACCGATTG_3′	134	57ºC–53ºC	54ºC	95ºC 15 min.	
**D16S3025**	204625	68532493	68532600	5′_TCCATTGGACTTATAACCATG_3′<br>5′_AGCTGAGAGACATCTGGG_3′	90-110	57ºC–53ºC	54ºC	95ºC 30sec.	
**rs16260 (C/A)**	94	68737131	x	5′_AACCGTGCAGGTCCCATAAC_3′<br>5′_CAAGCTCACAGGTGCTTTGC_3′	252	n.a.	57ºC		
CDH1		**68737225**	**68835548**					Touchdown<br>1 min. 30sec.	
**rs1801552 (C>T)**	Inside gene	68823538	x	5′_ TGGCCTTAGAGGTGGGTGAC_3′<br>5′_CCAGGAAATAAACCTCCTCCAT_3′	262	n.a.	55ºC		
**D16S496**	79259	68914807	68915022	5′_ GTTTGGCAGAAAGGCTACTT_3′<br>5′_GCCCATCCTCCACTATCTAT_3′	216	57ºC–53ºC	54ºC	72ºC 1 min.<br>30 sec	x nr cycles depending on Touchd. range
**D16S3067**	239217	69074765	69074908	5′_GCCACCTCACACTAGCCTG_3′<br>5′_TCACTCAAAATGGAGTCACTCTG_3′	138 - 152	n.a.	57ºC		
**D16S3095**	1076779	69912327	69912484	5′_TCAGTTGGAAGATGAGTTGG_3′<br>5′_TATAGTTTGTGTCCCCCGAC_3′	134 - 162	n.a.	55ºC	95ºC 30<br>seconds	
**D16S3106**	3318313	72153861	72154060	5′_GAGACCTACAGTCTTTTGCATTAC_3′<br>5′_TTTTGAAGCTGAGCAGAAGG_3′	166 - 206	n.a.	58ºC	Ta 1 min. 30<br>sec	
**D16S3018**	5303255	74138803	74139062	5′_GGATAAACATAGAGCGACAGTTC_3′<br>5′_AGACAGAGTCCCAGGCATT_3′	244-270	61ºC–55ºC	55ºC	72ºC 1 min.<br>30 sec	x 35 cycles maximum
**D16S3049**	10051895	78887443	78887697	5′_GCAATGAAGGCAACAAAGT_3′<br>5′_TTAAAAGACCTGGGGGAAT_3′	233 - 255	55ºC–53ºC*	53ºC	72ºC 10 min.<br>4ºC +∞	
**D16S3098**	12579243	81414791	81414947	5′_TTCCACACATAAGGTGAGTTT_3′<br>5′_TTGTCTGCTTCTTTACGGA_3′	151 - 165	55ºC–53ºC*	53ºC		

Footnote: n.a. (not applicable)

Supplemental Table S4.

Chromosomes' haplotypes from HDGC families used for TMRCA estimation.

		D16S514	D16S318	D16S3025	c.1901C>T	D16S496	D16S3067	D16S3095	D16S3106	D16S3049	D16S3098	DHSMAP Analysis
c.1901c>T Carriers	F7	2	4	1	2	1	5	9	2	5	2	A – Conservative Analysis
	F1	2	4	1	2	1	5	6	1	6	5	
	F2	2	4	1	2	1	5	6	1	6	5	
	F3	1	4	1	2	1	5	6	1	6	5	
	F7-A	1/3	4	1	2	1	5	9	2	5	2	B – Conservative Analysis + Inclusion of two derived haplotypes
	F2-F	1/5	4	1	2	1	5	6	1	6	5	
Controls	F7 – C1	3	6	8	1	1	6	5	1	5	2	Used in Analysis A and Analysis B
	F7 – C2	7	1	1	1	6	9	6	5	6	2	
	F7 – C3	6	1	3	1	6	8	6	1	5	6	
	F7 –C5	2	5	7	1	6	7	5	4	0	0	
	F1 - C2	2	5	8	1	6	9	9	1	3	5	
	F1 – C3	2	5	8	1	6	8	6	1	5	2	
	F1 – C4	5	4	8	1	6	7	6	2	4	5	
	F1 – C5	7	4	7	1	6	9	9	1	7	5	
	F1 –C6	7	5	8	1	6	9	9	1	7	6	
	F1 – C7	7	5	8	1	6	7	9	1	6	4	
	F2 –C2	5	1	9	1	6	7	9	2	6	2	
	F2 – C4	6	5	8	1	1	5	9	5	6	2	
	F2 – C3	1	5	4	1	6	8	1	2	1	4	
	F2 – C6	5	5	8	1	1	8	6	1	5	5	
	F3 – C1	1	5	8	1	6	9	6	4	6	5	
	F3 – C3	7	5	1	1	1	5	9	2	6	2	
	F3–C1,1	1	8	7	1	1	5	6	1	4	5	
	F3 - C5	7	7	1	1	6	9	6	4	5	1	

For the purpose of TMRCA calculation in DHSMAP software, different alleles were label as numbers. For the c.1901 C>T variant the C allele was label as 1 and T allele as 2. For the remaining STR markers the labels were based in the number of repeats, where the allele with the lower bp size was numbered as 1 and the others with larger bp sizes numbered 2, 3, 4 … n according with the corresponding mutational steps (e.g. D16S514: 116 bp allele – 1; 118 bp allele – 2; 120 bp allele 3; etc.); 0 means missing data.
