# Supplementary figures and images for: The CDH1 c.1901C>T Variant: A Founder Variant in the Portuguese Population with Severe Impact in mRNA Splicing
Source: Cancers (Basel). 2021 Sep 4;13(17):4464. doi: 10.3390/cancers13174464 (PMC8430675; doi:10.3390/cancers13174464)

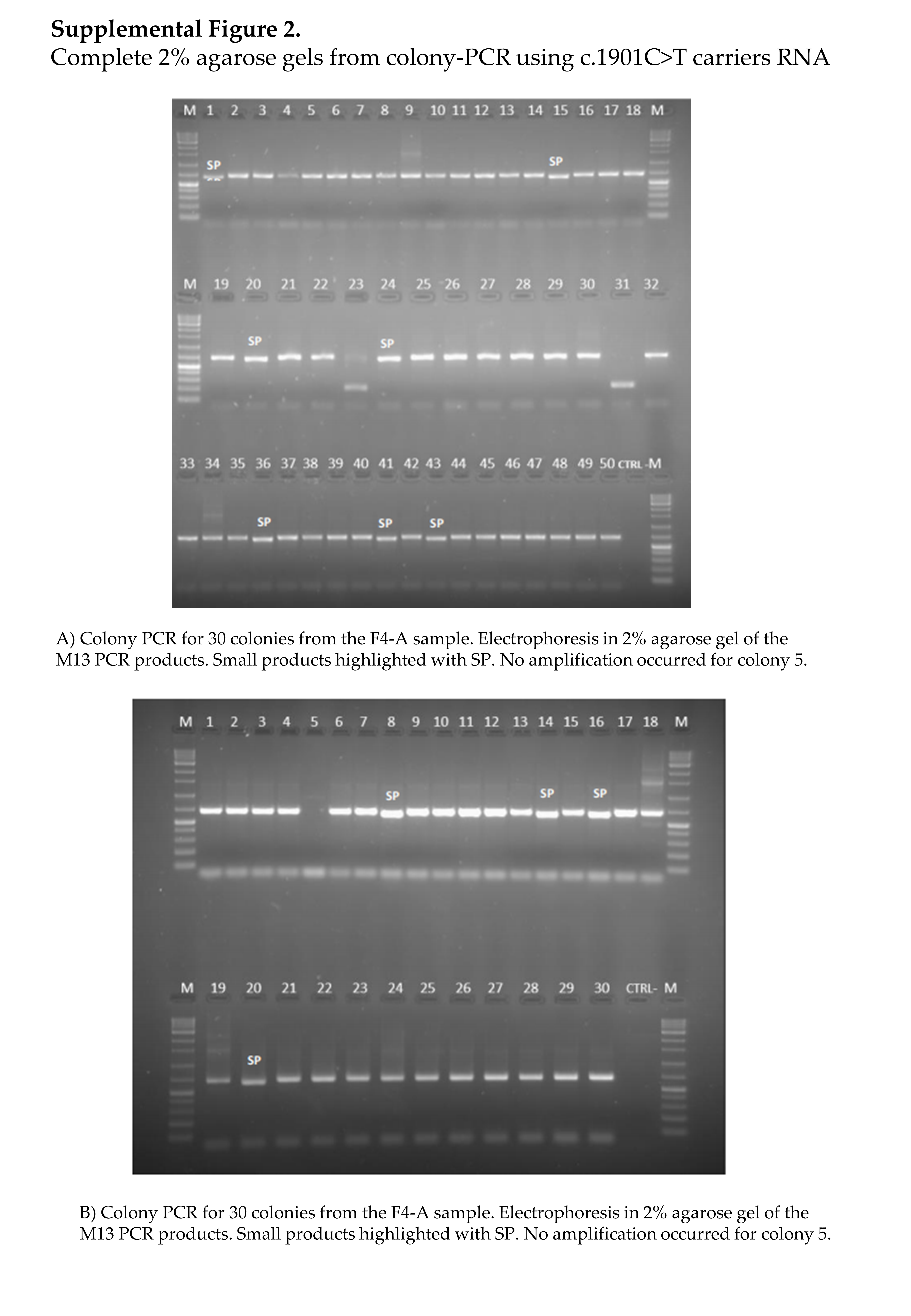

Supplement: Supplementary file 1 [file cancers-13-04464-s001.zip › Figures/Supplemental_Figure_S2.png]

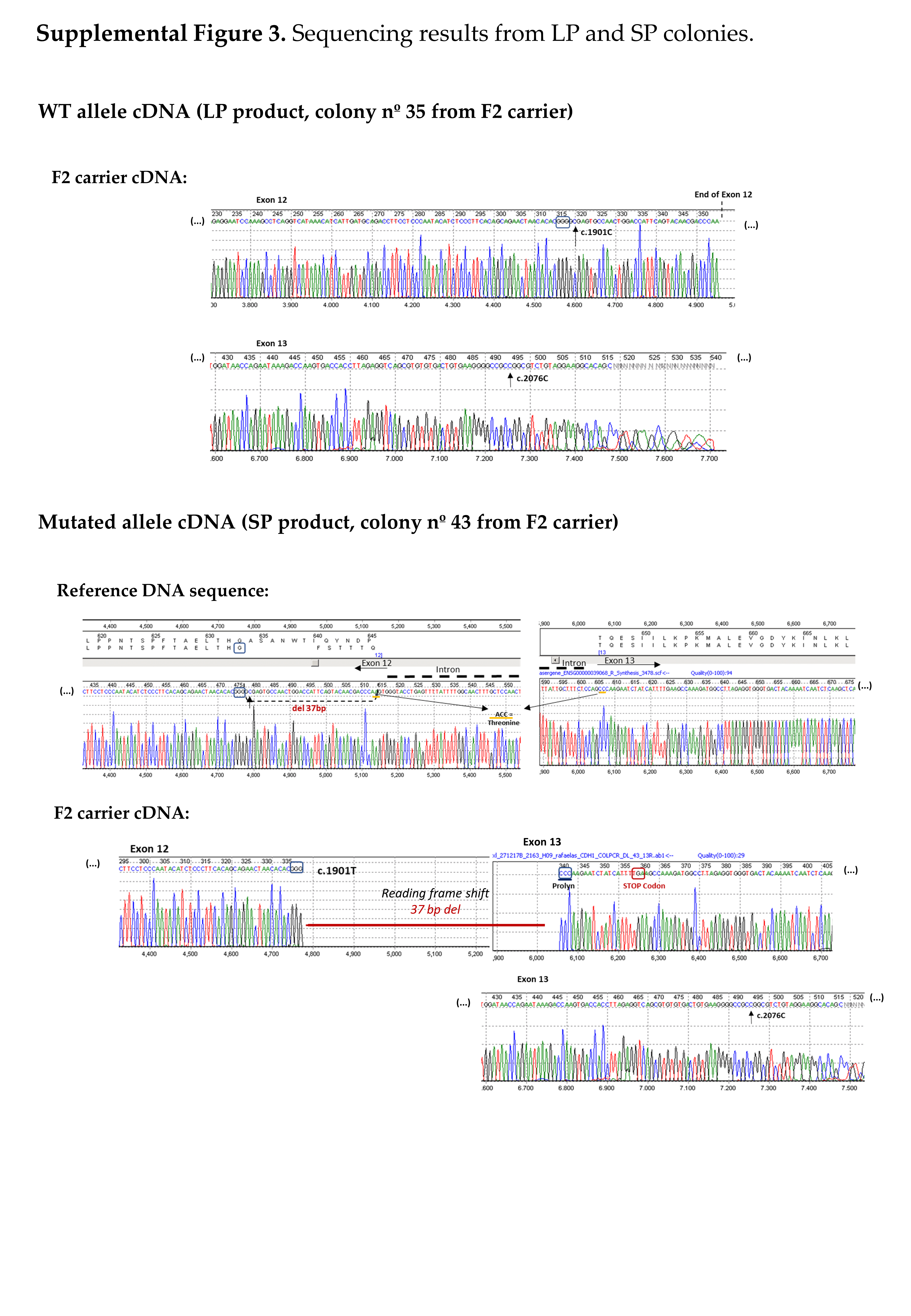

Supplement: Supplementary file 1 [file cancers-13-04464-s001.zip › Figures/Supplemental_Figure_S3.png]
